# A prospective study of real-time identification of line of transection in robotic colorectal cancer surgery by ICG

**DOI:** 10.1007/s11701-020-01095-2

**Published:** 2020-06-30

**Authors:** S. P. Somashekhar, Gangasani Revanth Kumar Reddy, Abhinav Yeshwant Deshpande, K. R. Ashwin, Rohit Kumar

**Affiliations:** 1 Aware Global Hospitals, Hyderabad, India; 2 Manipal Hospitals, Bangalore, India; 3 National Cancer Institute, Nagpur, India

**Keywords:** Colorectal cancer, Anastomotic leak, Bowel perfusion, ICG

## Abstract

Colorectal cancer is the second most common cancer in women and the third most common cancer in men in the world. Surgical resection is the gold standard treatment and minimally invasive surgery remains the standard of care. Anastomotic leakage is one of the most feared postoperative complications in colorectal surgery. Although several factors have been identified as possible causes of anastomotic leakage (i.e., surgical techniques, patient risk factors, suture material or devices), the complete pathogenesis is still unclear. The reported leak rate ranges from 1 to 30% and increases as the anastomosis is more distal. To date the most widely used methods to assess tissue perfusion includes the surgeon intraoperative visual judgement based on the colour; bleeding edges of resected margins; pulsation and temperature, thereby resulting in either excess or insufficient colonic resection. Earlier studies in colorectal surgery have suggested that assessment of tissue perfusion by the clinical judgment of the operating surgeon underestimated the risk of anastomotic leakage. Indocyanine green (ICG) is a intravenous dye which has shown promise in identifying the bowel vascularity real time. Earlier studies on colorectal cancer have shown that ICG based detection of bowel vascularity is technically possible and has reduced the anastomotic leak rates in 16.7% of patients. We conducted a prospective study on patients with ICG guided bowel perfusion during robotic colorectal cancer surgery. The method is technically easy, reproducible and safe. This technique has changed the intraoperative decision in 88% of patients. Larger studies are needed before this can become the standard of care.

## Background

Colorectal cancer is the second most common cancer in women and the third most common cancer in men in the world [1]. The clinical spectrum is different in India. The mean age at diagnosis is 47 years with male preponderance [2]. Most of the patients have advanced stage at presentation and Signet ring cell histology is predominant [3].

Surgical resection is the gold standard of treatment for colorectal cancer and minimally invasive surgery remains the standard of care [4]. Anastomotic leakage is one of the most feared postoperative complications in colorectal surgery with a reported leak rate ranges from 1 to 30% and increases as the anastomosis is more distal [5, 6].The reported rate of patients with an anastomotic leak that requires surgical revision ranges from 10 to 35% with a mortality rate ranging from 6 to 22% [7].

Although several factors have been identified as possible causes of anastomotic leakage (i.e., surgical techniques, patient risk factors, suture material or devices), the complete pathogenesis is still unclear [8] Poor local tissue oxygenation secondary to inadequate anastomotic vascular perfusion seems to play a key role in the determination of anastomotic viability.

The most widely used method to assess perfusion is a subjective clinical judgement by the surgeon based on pulsation, temperature and bleeding edges resulting in either excess or insufficient colonic resection. However, at least two studies [9, 10] have suggested that the clinical judgment of the operating surgeon underestimated the risk of anastomotic leakage in colorectal surgery.

Many methods of assessing vascularity like pulse oximetry, doppler ultrasound of marginal arteries, Laser doppler flowmetry, ultraviolet fluorescence studies, bowel wall contractility, rapid sampling microdialysis were used but with limited usage due to lack of reproducibility and practicality [11, 12]. Indocyanine green (ICG) is a new dye which has shown promise in identifying colonic vascularity [13]. This is a sterile, anionic, water-soluble but relatively hydrophobic, Tri carbocyanine molecule and, once injected into the vascular system, binds to plasma proteins [14].

It becomes fluorescent when excited by near-infrared light and has shown to identify the real-time image of vascularity of colon with a line of transection, thereby decreasing the risk of anastomotic leak and unnecessary excess colonic mobilization. The da Vinci robotic surgical system is integrated with fluorescence imaging (firefly technology). This provides real-time identification of anatomical structures using near-infrared imaging. ICG dye when injected into the tissues binds to plasma proteins and emits an infrared signal when excited by laser light. The camera of the endoscope has an infrared excitation laser (800 nm) and also has the ability to visualize infrared light(830 nm) [15].

Earlier studies in colorectal cancer have shown that ICG based detection of bowel vascularity has changed the plan of extensive bowel resection in 30% to 50% of patients and also significantly reduced the anastomotic leak rates [16, 17]. We conducted a single institutional prospective study to identify the feasibility of ICG identification of colonic vascularity and the change of plan of surgical management.

## Material/methods

This was a prospective study done on 50 patients with colorectal cancer satisfying the inclusion criteria and exclusion criteria over a period of twelve months. Patients with biopsy-proven colorectal cancer and Colorectal cancer patients post Neoadjuvant chemoradiation were included in the study with the exclusion of patients with metastatic colorectal cancer and Pregnant or lactating women and patients with known allergy to ICG.

All the patients underwent standard robotic surgery. The inferior mesenteric artery was fully dissected and ligated at its origin preserving the nerves. We chose high ligation because of better lymph node harvest and complete mobilization for resection. There is no unified consensus on the site of ligation and we have followed this in our study for low anterior resection patients. Colonic mobilisation was done in a “medial-to-lateral” approach with high ligation of the main feeding vessels was done. Stapled anastomosis was intra-corporeal in all the cases. After distal transection of the bowel, the point of proximal transection was judged by the team of 3 people consisting of the operating surgeon, assistant surgeon and a fellow in robotic surgery. The point of transection was identified in the vascular segment well above the margin of 5cms from tumor edge. This point was labelled B. ICG was then injected (3 ml of 2.5 mg/ml) intravenously and then line of demarcation identified in firefly mode and labelled point A if moved more than 5 mm proximal to the original and point C if moved more than 5 mm distally. Any change less than 5 mm remained as point B. The perfusion images were recorded and assessed in real time.

## Results

The results of the study are represented in the flow diagram which shows the total number of patients enrolled in the study were 65 in number of which 15 were excluded and 50 patients were included for the final study.
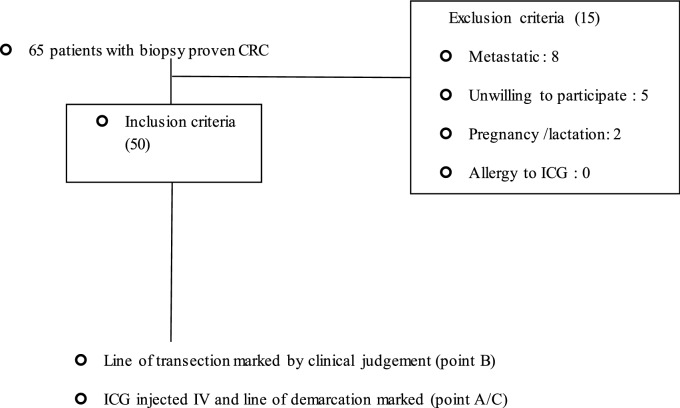


**Fig. 1:**
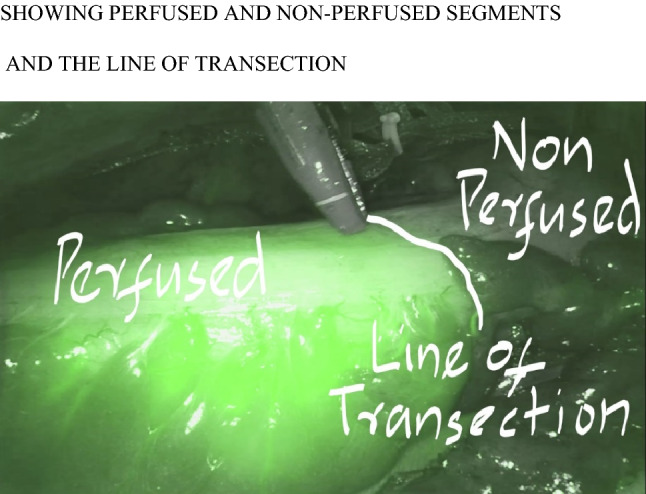
Showing perfused and non-perfused segments and the line of transection

**Fig. 2:**
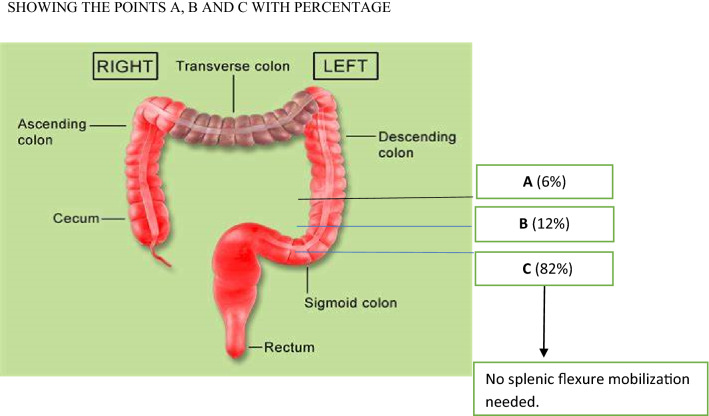
Showing the points a, b and c with percentage

Firefly technology was used to assess bowel perfusion in all patients.The patient characteristics in the study are shown in Table1 which shows the mean age of patients was 54.52 ± 12.6 years and there were 18 females and 32 males (Figs. 1, 2).

**Table 1:** Showing patient demographics

Patient charecteristics	No. of patients
Age (years) Median ± SD	54.52 ± 12.6 years
Male	32
Female	18
Location	
Colon	6
Rectum	44
TNM staging	
I	0
II	3
III	47
IV	0
Operative procedure	
Sigmoidectomy	6
Low anterior resection	44
Illeostomy	44
Pre-operative chemoradiation	44
High ligation of IMA	44
Splenic flexure mobilisation	44

All the rectal cancer patients had a locally advanced disease and received preoperative chemoradiation. 47 patients had stage 3 disease and 3 sigmoid colon patients had stage 2 disease. Six patients had a sigmoid colectomy and 44 patients underwent low anterior resection with sigmoid colon mobilization and ileostomy. There were no intraoperative adverse events or conversion to open surgery. Protective ileostomy was carried out in all low anterior resection patients. There were no side effects related to the injection of ICG. As shown in Table 2, ICG-enhanced fluorescence was detected in 100% of the cases.

**Table 2:** Showing the results of the study

Total no of cases	50
Identification	50
Line of transection moved proximally (> 5 mm change)	3
Line of transection moved distally (> 5 mm change)	41
Line of transecteion remained the same (< 5 mm change)	6
Change of plan	44
Conversion to open	0
Anastomotic leakage	0

41 patients had a change of plan to distal transection and 3 patients had a change of plan to proximal resection with more than 5 mm change. Only 6 patients had no change in the surgical plan. There were no anastomotic leaks in all the patients who underwent a change in surgical plan based on intraoperative perfusion assessment by ICG. Reversal of ostomy was performed in the patients after endoscopic evaluation.

## Discussion

Anastomotic failure is the most feared complication of bowel surgery. Vascular perfusion is pivotal for anastomotic integrity of the bowel. Adequate vascular supply of the left colon depends on the patency of the inferior mesenteric artery, the left colic artery and also relies on patency of the middle colic artery and the marginal artery of Drumond and eventually Riolan arcades [24, 25]. Anatomic variations are frequent, and aberrations such as the absence of the middle colic artery or inadequate vascularization of the splenic flexure are frequent (up to 25%) [26–29].

The vascular perfusion of bowel can be assessed subjectively and objectively. Subjective assessment by visual inspection under white light has been the gold standard and is widely practiced. This method of visual inspection alone has shown to underestimate the vascularity in previous studies [18–20] leading to the removal of either excess colon or less colon. Many methods of objective assessment of bowel perfusion are available which lack reproducibility and are cumbersome [30]. ICG is a intravenous dye which can identify the vascularity of bowel real-time objectively.

Indocyanine green angiography is a more recent development in colorectal surgery. Previous studies in literature by Kawada et al. have shown a 30% change of plan with ICG and Hellen et al. have shown a 50% change of plan. Kudszus et al. [21] demonstrated a reduction in risk of surgical revision of 60% (7.5–3.5%) using fluorescence angiography in a case-matched retrospective study. These data have been confirmed by Jafari et al. [22] during robotic-assisted laparoscopic rectal surgery.

A recent retrospective case-matched study by Kin et al. [23] on colorectal resection demonstrated that once fluorescence angiography was performed, surgeons decided to change the proximal resection margin alone in 8/173 patients (4.6%) but this was not correlated with a significant reduction in the leak rate once compared to the matched group where fluorescence angiography was not performed.

The effect of repeated injections of ICG is not known and has not been investigated [31]. Another drawback is that there is still no strict analytic measure to objectively quantify the signal intensity, and the evaluation of images still depends on the surgeon’s judgment.

At least two studies [32, 33] focused on the blood supply assessed using Doppler flowmetry, but although both demonstrated reduced blood flow after resection, it is not clear what is the minimal blood flow to avoid anastomotic leakage [20, 34]. Despite the limitations, ICG-enhanced fluorescence seems to be safe, reproducible, and cost-effective to assess colonic and anastomotic perfusion [35].

## Conclusion

As per our experience, the injection of ICG provides real-time identification of bowel perfusion after vascular division and shows the line of demarcation between vascular and avascular segments. It has changed the intraoperative decision in 88% of our patients and amongst whom splenic flexure mobilization could have been avoided in 82% of patients. It is technically easy, reproducible and safe and larger studies are needed to provide evidence for its routine use in robotic rectal cancer surgery.
